# Patterns and predictors of first and subsequent recurrence in women with early breast cancer

**DOI:** 10.1007/s10549-017-4340-3

**Published:** 2017-07-04

**Authors:** Y. M. Geurts, A. Witteveen, R. Bretveld, P. M. Poortmans, G. S. Sonke, L. J. A. Strobbe, S. Siesling

**Affiliations:** 1 Department of Health Technology and Service Research, MIRA Institute for Biomedical Technology and Technical Medicine, University of Twente, Postbus 217, 7500 AE Enschede, The Netherlands; 2 Department of Research, Netherlands Comprehensive Cancer Organisation, Postbus 217, 7500 AE Enschede, The Netherlands; 3 Department of Radiation Oncology, Radboud University Medical Center, Geert Grooteplein Zuid 10, 6525 GA Nijmegen, The Netherlands; 4 Department of Medical Oncology, Netherlands Cancer Institute, Postbus 90203, 1006 BE Amsterdam, The Netherlands; 5 Department of Surgical Oncology, Canisius-Wilhelmina Hospital, Weg door Jonkerbos 100, 6532 SZ Nijmegen, The Netherlands

**Keywords:** Breast cancer, Recurrence patterns, Recurrence risk, Follow-up, Prognostic factors

## Abstract

**Purpose:**

Little is known about the occurrence, timing and prognostic factors for first and also subsequent local (LR), regional (RR) or distant (DM) breast cancer recurrence. As current follow-up is still consensus-based, more information on the patterns and predictors of subsequent recurrences can inform more personalized follow-up decisions.

**Methods:**

Women diagnosed with stage I-III invasive breast cancer who were treated with curative intent were selected from the Netherlands Cancer Registry (*N* = 9342). Extended Cox regression was used to model the hazard of recurrence over ten years of follow-up for not only site-specific first, but also subsequent recurrences after LR or RR.

**Results:**

In total, 362 patients had LR, 148 RR and 1343 DM as first recurrence. The risk of first recurrence was highest during the second year post-diagnosis (3.9%; 95% CI 3.5–4.3) with similar patterns for LR, RR and DM. Young age (<40), tumour size >2 cm, tumour grade II/III, positive lymph nodes, multifocality and no chemotherapy were prognostic factors for first recurrence. The risk of developing a second recurrence after LR or RR (*N* = 176) was significantly higher after RR than after LR (50 vs 29%; *p* < 0.001). After a second LR or RR, more than half of the women were diagnosed with a third recurrence.

**Conclusions:**

Although the risk of subsequent recurrence is high, absolute incidence remains low. Also, almost half the second recurrences are detected in the first year after previous recurrence and more than 80% are DM. This suggests that more intensive follow-up for early detection subsequent recurrence is not likely to be (cost-)effective.

**Electronic supplementary material:**

The online version of this article (doi:10.1007/s10549-017-4340-3) contains supplementary material, which is available to authorized users.

## Introduction

As a result of early detection and improved treatment, survival after breast cancer has improved significantly. Consequently, an increasing number of women is in need of follow-up care after curative treatment [1]. The main aim of follow-up is the early detection of local (LR) or regional recurrences (RR) and secondary primary tumours [2]. The incidence of first recurrence is influenced by prognostic factors such as age, grade, nodal involvement, hormone receptor status and treatment of the primary tumour [3, 4]. Patients with LR or RR have a higher risk of developing distant metastasis (DM) and have worse survival compared to patients without LR or RR [5–9]. Information on the pattern of site-specific first and second recurrence after LR or RR is lacking and the effect of prognostic factors such as the disease-free interval (DFI) after a first LR or RR on the development of subsequent recurrences is not well documented. Earlier studies showed a peak in first recurrence hazard approximately two years after the primary tumour [10–12]. Some studies also demonstrated a second peak between 4–9 years after treatment, mainly in ER-positive patients [10–16]. However, these studies lack contemporary treatments [11] or report single-institution data [12–16]. Also the pattern of subsequent recurrence after the first is not yet analysed.

The pattern of recurrence risk and prognostic factors for the development of subsequent breast cancer recurrences can provide valuable information for informed clinical decision-making and patient centred follow-up. Using a population-based cohort of women with follow-up data of ten years after treatment for primary invasive breast cancer, we aimed to (1) analyse the occurrence and timing of not only first, but also subsequent LR, RR and DM, (2) identify prognostic factors for first and subsequent LR, RR and DM and (3) identify consequences of these patterns for tailoring of follow-up.

## Patients and methods

### Data collection and study population

Data originate from the nationwide population-based Netherlands Cancer Registry (NCR), for which data are collected directly from patient medical records by a team of specially trained data managers. All data are registered in accordance with national and international coding rules, and include patient, primary tumour and treatment characteristics, as well as data concerning tumour recurrences within ten years following diagnosis.

Women with primary invasive breast cancer diagnosed in Dutch hospitals in 2003 were included (*N* = 10,356). Eligibility criteria were stage I–III breast cancer, no previous or synchronous cancer, no direct extension to chest wall or skin, surgical treatment in a Dutch hospital and no neo-adjuvant therapy. Patients with macroscopic residue after surgical treatment or microscopic residue without adjuvant treatment were excluded.

### End-points

Site of recurrence was classified as follows: (1) local—any epithelial breast cancer or ductal carcinoma in situ (DCIS) in ipsilateral breast tissue, or in skin and subcutaneous tissue of the ipsilateral thoracic wall, (2) regional—breast cancer in ipsilateral lymph nodes or contralateral lymph nodes if axillary lymph node dissection was performed, or (3) distant—breast cancer in any other location except the contralateral breast [17].

Only patients with LR or RR as first recurrence were considered at risk for subsequent LR, RR or DM. Subsequent recurrences after DM were not taken into account, as DM is considered incurable and further LR or RR has no consequence. For patients with synchronous recurrences, the most life-threatening site of recurrence was taken as endpoint: in case of synchronous LR and RR, the recurrence was registered as RR; when DM was diagnosed with LR or RR, DM was registered. Within the NCR, tumours detected within three months after diagnosis of the previous tumour were considered synchronous. Therefore, follow-up time started three months after diagnosis of the primary tumour. The time to first recurrence or disease-free interval (DFI) was measured from three months after diagnosis of the primary tumour to date of detection of recurrence at any site. DFI to second and third recurrence was measured from detection of the previous recurrence to detection of subsequent recurrence. Follow-up time was censored at ten years after start of follow-up. In addition, survival analyses were performed per type of recurrence for survival after the primary tumour, after first and after second recurrence.

### Prognostic factors

Variables were selected based on literature and availability of the data. Estrogen receptor (ER) and progesterone receptor (PR) status were combined into one prognostic factor (ER or PR positive, ER and PR negative) [18]. Age at diagnosis of the primary tumour (<40, 40–49, 50–75, >75), size (≤2, >2–5, ≥5 cm), grade (I, II, III), histological type (ductal, lobular or other), multifocality (yes, no), lymph node status (negative, 1–3, 4–9 nodes), hormone receptor status, type of surgery (breast conserving (BCS) or mastectomy), chemo-, endocrine and radiation therapy (all yes or no) of the primary tumour were assessed as prognostic factors for first recurrence. The same patient, primary tumour and treatment characteristics, DFI and treatment of the recurrence (surgery, chemo-, radiation, and endocrine therapy) were analysed as prognostic factors for subsequent recurrence.

### Statistical analysis

The number of recurrences at specific recurrence sites were compared using the Wilcoxon rank sum test. We identified prognostic factors for site-specific first recurrence and subsequent recurrences after LR or RR using extended Cox regression analysis. A minimum hazard ratio (HR) of 1.1 was used for inclusion in the multivariable analysis. To avoid overfitting, an effective sample size of ten per estimated parameter was used, excluding the ones with the smallest effect first [19]. Variables that were significant when testing the proportional hazards assumption by means of Schoenfeld residuals and non-parallel on the log–log plot of the DFI were included as time-varying coefficients in the extended Cox model. Cox regression was also used for modelling the survival after the primary tumour and the first and second recurrence. With the logrank test, the equality of the survival functions was tested.

Prognostic factors with missing values were multiple imputed using a chained equation approach [20]. It was assumed that missing values occurred at random, which validated the use of imputation. All tests were two-sided, and probability values of <0.05 were considered statistically significant. All analyses were performed in STATA^®^ 14.0.

## Results

After exclusion, 9342 patients were included in the analyses. Table 1 summarizes the characteristics of the study population. Median age at diagnosis was 58 years (range 20–96). The majority of patients had primary tumours ≤2 cm (60%), grade II disease (44%) and no nodal involvement (61%). Median follow-up was ten years (interquartile range (IQR) 6.5–10.0), in which 27% of the patients died. Forty imputed datasets were pooled using Rubin’s rules and showed healthy convergence.

**Table 1:** Patient, tumour and primary treatment characteristics categorized by subsequent recurrence

Characteristic	No recurrence (*N* = 7489)		First recurrence DM (*N* = 1343)		First recurrence LR/RR (*N* = 510)		Second recurrence LR/RR (*N* = 52)		Total (*N* = 9342)	
	*N*	%	*N*	%	*N*	%	*N*	%	*N*	%
Age at diagnosis										
<40	410	5.5	126	9.4	57	11.2	3	5.8	593	6.4
40–49	1455	19.4	293	21.8	106	20.8	6	11.5	1854	19.9
60–74	4625	61.8	749	55.8	296	58.0	37	71.2	5670	60.7
≥75	999	13.3	175	13.0	51	10.0	6	11.5	1225	13.1
Tumour size										
≤2 cm	4772	63.7	523	38.9	285	55.9	24	46.2	5580	59.7
2–5 cm	2484	33.2	728	54.2	201	39.4	24	46.2	3413	36.5
>5 cm	177	2.4	77	5.7	15	2.9	3	5.8	269	2.9
Unknown	56	0.8	15	1.1	9	1.8	1	1.9	80	0.9
Grade										
I	1660	22.2	96	7.2	91	17.8	3	5.8	1847	19.8
II	3006	40.1	534	39.8	187	36.7	22	42.3	3727	39.9
III	2122	28.3	602	44.8	180	35.3	25	48.1	2904	31.1
Unknown	701	9.4	111	8.3	52	10.2	2	3.9	864	9.3
Histology										
Invasive ductal	5948	79.4	1096	81.6	408	80.0	43	82.7	7452	79.8
Invasive lobular	801	10.7	158	11.8	57	11.2	6	11.5	1016	10.9
Other	740	9.9	89	6.6	45	8.8	3	5.8	874	9.4
Lymph nodes										
Negative	4772	63.7	509	37.9	318	62.4	24	46.2	5599	59.9
1–3 positive	1972	26.3	424	31.6	138	27.1	21	40.4	2534	27.1
4–9 positive	607	8.1	401	29.9	41	8.0	7	13.	1049	11.2
Unknown	138	1.8	9	0.7	13	2.6	0	0.0	160	1.7
Hormone status										
ER and PR−	1152	15.4	325	24.2	130	25.5	14	26.9	1607	17.2
ER/PR+	6025	80.5	960	71.5	366	71.7	38	73.1	7351	78.7
Unknown	312	4.2	58	4.3	14	2.8	0	0.0	384	4.1
Multifocality										
No	6269	83.7	1061	79.0	434	85.1	41	78.9	7764	83.1
Yes	693	9.25	187	13.9	46	9.0	6	11.5	926	9.9
Unknown	527	7.0	95	7.1	30	5.9	5	9.6	652	7.0
Surgery type										
BCS	4246	56.7	581	43.3	279	54.7	22	42.3	5106	54.7
Mastectomy	3243	43.3	762	56.7	231	45.3	30	57.7	4236	45.3
Microscopic residue										
No	7083	94.6	1231	91.7	479	93.9	50	96.2	8793	94.1
Yes	250	3.3	50	3.7	16	3.1	1	1.9	316	3.4
Unknown	156	2.1	62	4.6	15	2.9	1	1.9	233	2.5
Chemotherapy										
No	5057	67.5	660	49.1	350	68.6	34	65.4	6067	64.9
Yes	2432	32.5	683	50.9	160	31.4	18	34.6	3275	35.1
Radiation therapy										
No	2631	35.1	421	31.4	207	40.6	26	50.0	3259	34.9
Yes	4858	64.9	922	68.7	303	59.4	26	50.0	6083	65.1
Endocrine therapy										
No	4370	58.4	643	47.9	368	72.2	30	57.7	5381	57.6
Yes	3119	41.7	700	52.1	142	27.8	22	42.3	3961	42.4

*LR* local recurrence, *RR* regional recurrence, *DM* distant metastasis, *ER* oestrogen receptor, *PR* progesterone receptor, *BCS* breast conserving surgery

### Patterns of first recurrence

**Fig. 1:**
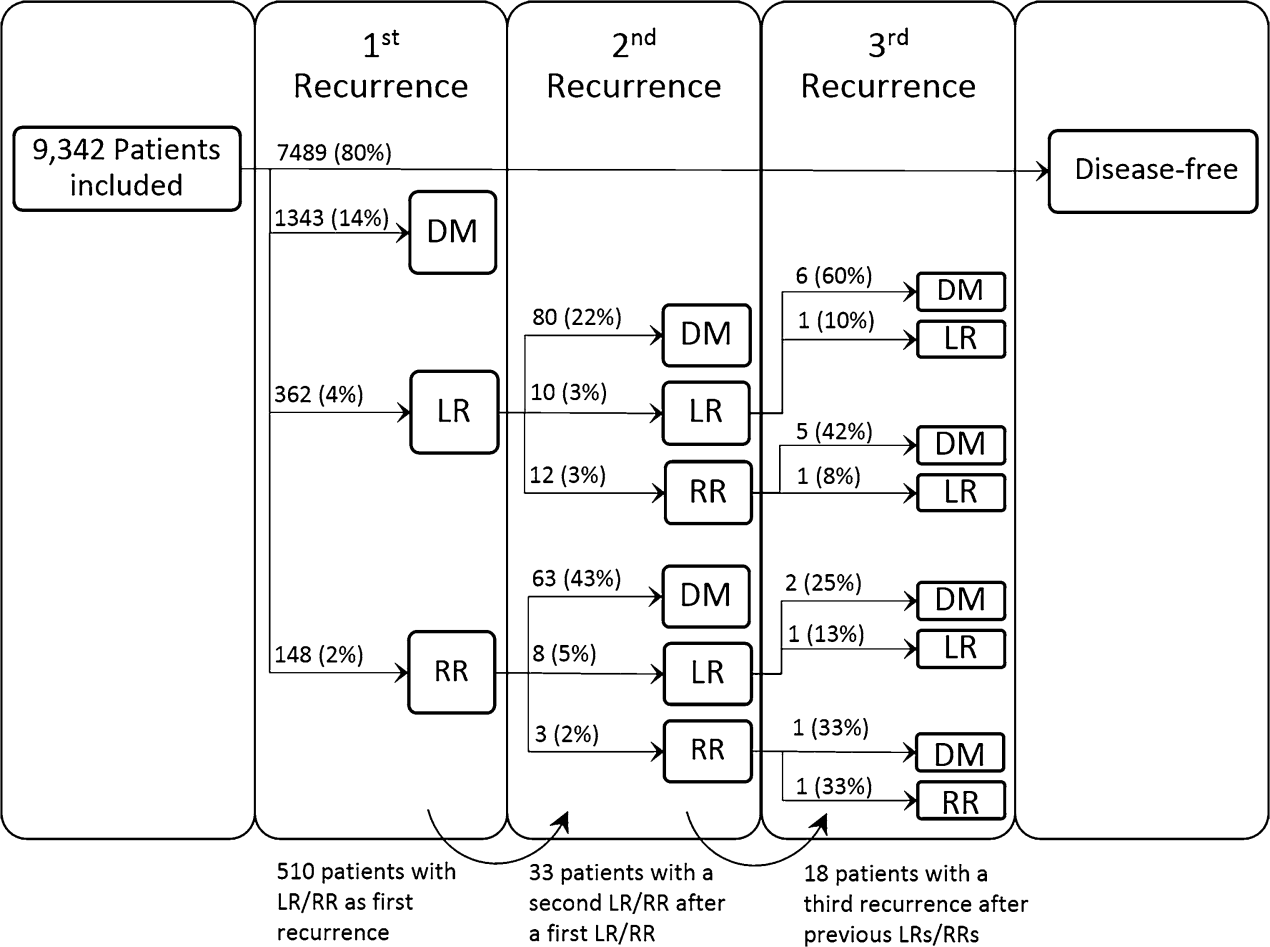
Flow chart of first and subsequent recurrences. Abbreviations: *LR* local recurrence, *RR* regional recurrence, *DM* distant metastasis

**Fig. 2:**
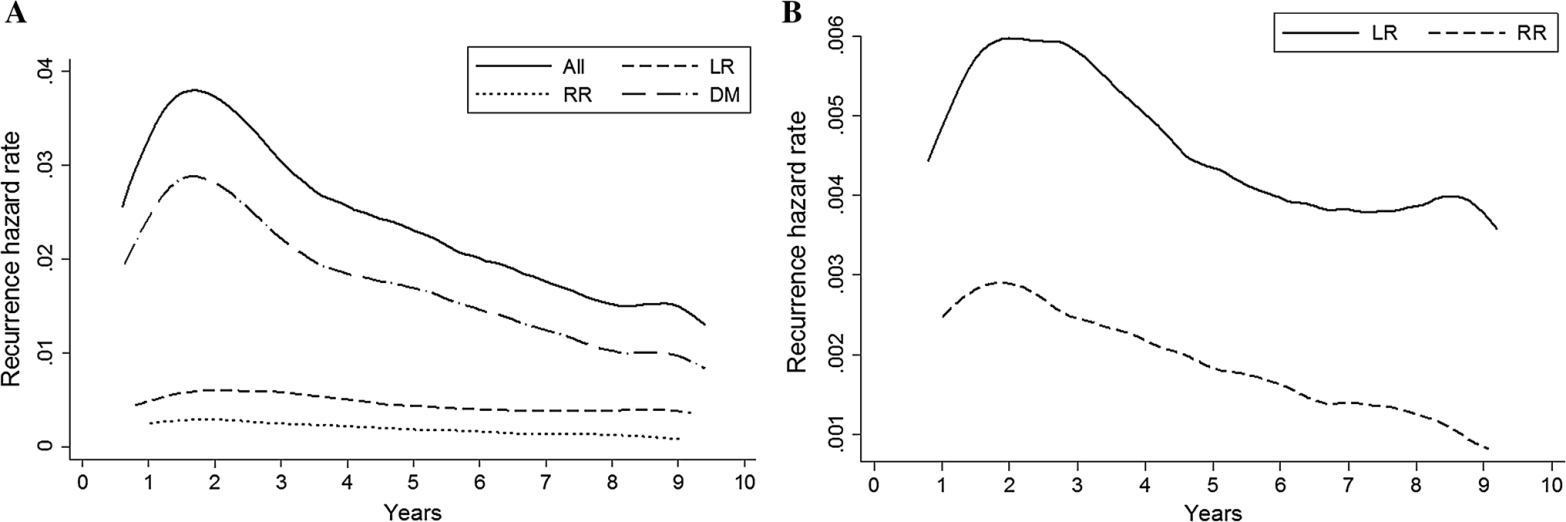
Hazard of **a** all first recurrences, and **b** first LR and RR during 10 years of follow-up. Abbreviations: *LR* local recurrence, *RR* regional recurrence, *DM* distant metastasis

Recurrence occurred in 1853 patients (20%) of which 362 patients (20%) had LR, 148 (8%) RR and 1343 (72%) DM (Fig. 1). For the entire cohort, the risk of recurrence was highest in the second year after diagnosis (3.9%; 95% CI 3.5–4.3), with a median DFI of 3.3 years (Fig. 2a). A second peak was present around year 8. However, as there was overlap in the 95% CIs, this finding was not statistically significant. For LR and DM separately, a similar risk pattern was present. The risk of DM was 3% (95% CI 2.6–3.3) during the second year, with a median DFI of 3.2 years. The annual risk of LR showed a double peaked pattern with a risk of 0.7% (95% CI 0.5–0.8) in year two and 0.4% (95% CI 0.2–0.5) in year eight. Median DFI was 3.6 years (Fig. 2b). The annual risk of RR showed a single peak of 0.3% (95% CI 0.2–0.4) in year two and decreased thereafter. Median DFI was 3.1 years (Fig. 2b). Patients with a recurrence around year 8–9 (second peak) had on average a better differentiated primary tumour, positive hormone receptor status and received more often breast conserving therapy with radiation therapy, compared to patients with an early recurrence around year two. Supplementary Figures S1–12 illustrate the patterns of first recurrence stratified by the different prognostic factors. The peak in risk around year two was most pronounced for patients with grade III tumours (Fig. S4) or negative hormone status (Fig. S5), while the pattern of first recurrence showed a more gradual decline in patients with ER-positive tumours.

### Patterns of second and third recurrence

**Fig. 3:**
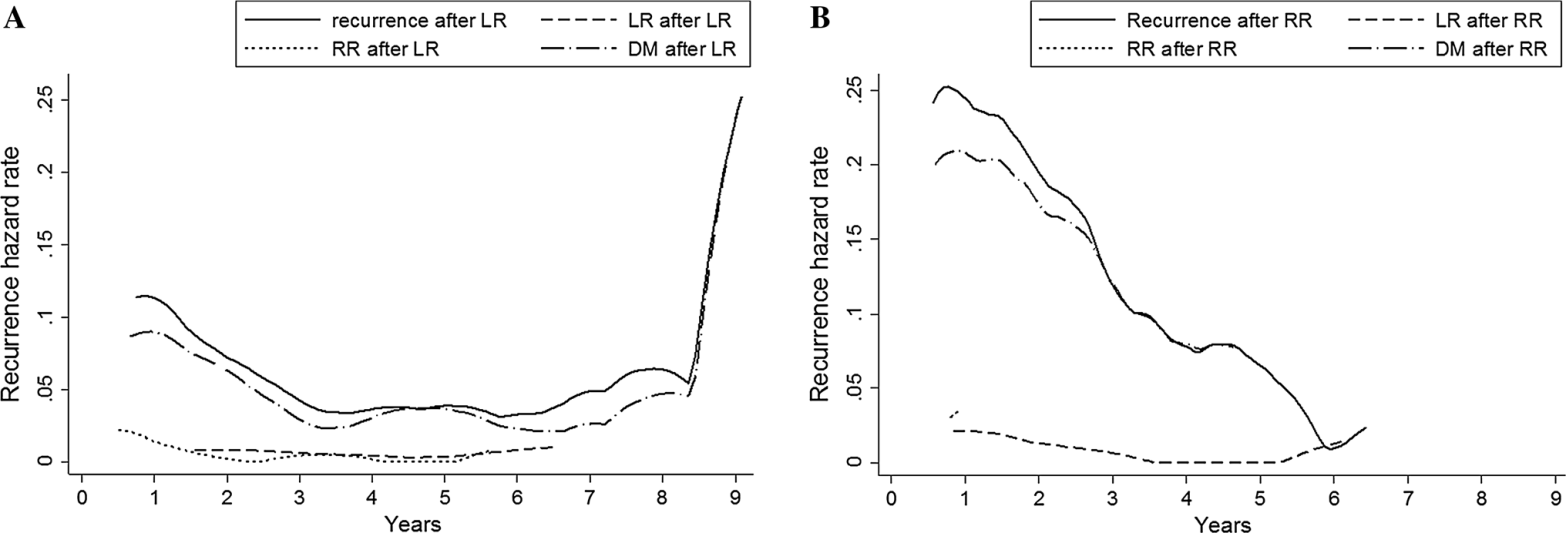
Hazard of subsequent (second) recurrence after **a** first LR, and **b** first RR. Abbreviations: *LR* local recurrence, *RR* regional recurrence, *DM* distant metastasis

After LR, 102 out of 362 patients (28%) developed a second recurrence (1.1% of total population). Of those, 10 patients had another LR (10%), 12 patients RR (12%) and 80 patients DM (78%). The risk of subsequent recurrence after LR reached its maximum of 15% (95% CI 11–20%) in the first year after previous LR (Fig. 3a). A second peak was present in the eighth year after previous LR (6.4%; 95% CI 0.0–13.6). However, as there were only 7 events after a previous LR after year 7, this finding was not statistically significant. Median DFI after LR was 1.1 year (IQR 0.3–2.5). Ninety-five percent of all subsequent recurrences after first LR occurred between 5 weeks and 7 years.

Seventy-four out of 148 patients (50%, 0.8% of total population) had subsequent recurrence after first RR; eight patients had LR (11%), three patients had another RR (4%) and 63 patients DM (85%). The proportion of patients with recurrence after RR was significantly higher than the proportion of patients with recurrence after LR (*p* < 0.001). The risk of second recurrence after RR was highest in the first year (2.7%; 95% CI 1.8–3.6) (Fig. 3b). Median DFI after RR was 1.1 year (IQR 0.6–2.2). After RR, 95% of all subsequent recurrences occurred between 6 weeks and 5 years after diagnosis of the first.

Eighteen patients (55%, 0.2% of the total population) with two previous LR or RR (*N* = 33) developed a third recurrence. Seven patients (39%) had two previous LRs, two patients (11%) had two previous RRs and nine patients (50%) had previous LR and RR.

### Prognostic factors for first and subsequent recurrences

In univariable analysis, age at diagnosis, size and grade of the primary tumour, lymph node status, multifocality and chemotherapy were statistically significant prognostic factors for first recurrence. Multivariable analysis demonstrated that >3 positive lymph nodes was the most important prognostic factor for occurrence of overall first recurrence (HR 3.7, compared to N0), followed by age <40 years and tumour size >5 cm. Younger age, negative hormone status, BCS and no endocrine therapy were most influential factors for first LR, and besides these, also primary tumour size and radiation therapy for first RR (Table 2). Tumour grade II/III and >3 positive lymph nodes displayed the highest HRs for first DM. With 102 observed events after first LR and 74 observed events after first RR, the number of variables in the multivariable analyses for subsequent recurrence after LR and RR was set to ten and seven, respectively (Table 3). Prognostic factors for recurrence after LR were larger primary tumour size, positive lymph nodes and higher grade. Larger primary tumour size, multifocality and shorter DFI to first RR were prognostic factors for recurrence after RR. With only 33 patients at risk and 18 events, we did not have enough power to identify prognostic factors for a third recurrence.

**Table 2:** Prognostic factors for site-specific first recurrence during 10 years of follow-up in women with stage I–III invasive breast cancer (*n* = 9342)

Characteristic	LR (362 events)						RR (148 events)					
	Univariable			Multivariable			Univariable			Multivariable		
	HR	95% CI	*p*	HR	95% CI	*p*	HR	95% CI	*p*	HR	95% CI	*p*
Age (years)												
<40	Ref			Ref			Ref			Ref		
40–49	0.67	0.45–0.99	0.045	0.59	0.40–0.88	0.009	0.34	0.19–0.61	<.001	0.35	0.19–0.63	<.001
50–74	0.54	0.38–0.78	0.001	0.43	0.30–0.61	<.001	0.44	0.27–0.70	0.001	0.42	0.25–0.71	0.001
≥75	0.72	0.46–0.12	0.141	0.62	0.39–0.99	0.043	0.25	0.11–0.54	<.001	0.13	0.05–0.32	<.001
Tumour size												
≤2 cm	Ref						Ref			Ref		
2–5 cm	1.09	0.88–1.35	0.441				2.05	1.47–2.86	<.001	2.18	1.51–3.15	<.001
>5 cm	0.92	0.45–1.86	0.814				2.65	1.22–5.77	0.014	2.72	1.13–6.57	0.026
Grade												
I	Ref						Ref			Ref*		
II	0.95	0.72–1.26	0.731				1.69	0.96–2.99	0.069	*1.18*	*0.81*–*1.72*	*0.398*
III	1.05	0.79–1.39	0.751				3.35	1.94–5.77	<.001	*1.28*	*0.84*–*1.96*	*0.254*
Lymph nodes												
Negative	Ref			Ref*			Ref			Ref		
1–3 positive	1.04	0.82–1.32	0.738	*1.16*	*0.95*–*1.42*	*0.133*	0.95	0.65–1.39	0.796	0.86	0.57–1.29	0.464
>3 positive	0.91	0.62–1.34	0.626	*0.88*	*0.64*–*1.21*	*0.420*	0.89	0.49–1.62	0.693	1.00	0.51–1.96	0.999
Endocrine status												
ER&PR negative	Ref			Ref*			Ref			Ref*		
ER/PR positive	0.67	0.52–0.86	0.001	*1.59*	*1.27*–*2.00*	*0.001*	0.35	0.25–0.49	<.001	*0.85*	*0.61*–*1.20*	*0.364*
Histology												
Ductal	Ref			Ref			Ref			Ref*		
Lobular	1.17	0.85–1.60	0.329	1.21	0.88–1.66	0.246	0.69	0.38–1.24	0.213	*1.03*	*0.69*–*1.53*	*0.890*
Other	1.05	0.74–1.48	0.797	0.99	0.69–1.41	0.950	0.58	0.30–1.14	0.116	*0.85*	*0.53*–*1.37*	*0.505*
Multifocality												
No	Ref			Ref			Ref			Ref		
Yes	0.98	0.69–1.39	0.908	1.12	0.77–1.62	0.562	0.78	0.43–1.41	0.41	0.62	0.34–1.13	0.120
Surgery type												
Breast conserving	Ref			Ref*			Ref					
Mastectomy	0.87	0.70–1.08	0.197	*0.74*	*0.63*–*0.87*	<*.001*	2.17	1.56–3.02	<.001			
Residual disease												
No	Ref						Ref			Ref		
Microscopic	0.92	0.51–1.68	0.794				0.99	0.40–2.41	0.979	1.44	0.58–3.57	0.437
Chemotherapy												
No	Ref						Ref			Ref		
Yes	0.77	0.61–0.97	0.024				1.07	0.77–1.50	0.673	0.67	0.42–1.05	0.081
Radiation therapy												
No	Ref						Ref			Ref		
Yes	0.9	0.72–1.11	0.331				0.43	0.31–0.60	<.001	0.42	0.29–0.60	<.001
Endocrine therapy												
No	Ref			Ref*			Ref					
Yes	0.47	0.37-0.59	<.001	*0.62*	*0.51*–*0.75*	<*.001*	0.67	0.47–0.94	<.001			

Characteristic	DM (1343 events)						All (1853 events)					
	Univariable			Multivariable			Univariable			Multivariable		
	HR	95% CI	*p*	HR	95% CI	*p*	HR	95% CI	*p*	HR	95% CI	*p*
Age (years)												
<40	Ref						Ref			Ref*		
40–49	0.69	0.56–0.85	<.001				0.65	0.54–0.77	<.001	*0.83*	*0.73*–*0.95*	*0.007*
50–74	0.58	0.48–0.70	<.001				0.55	0.47–0.65	<.001	*0.84*	*0.74*–*0.96*	*0.008*
≥75	0.82	0.65–1.03	0.087				0.73	0.60–0.89	0.002	*0.81*	*0.68*–*0.96*	*0.016*
Tumour size												
≤2 cm	Ref			Ref*			Ref			Ref*		
2–5 cm	2.60	2.33–2.91	<.001	*1.27*	*1.16*–*1.39*	<*.001*	2.15	1.95–2.36	<.001	*1.22*	*1.14*–*1.32*	<*.001*
>5 cm	3.71	2.92–4.72	<.001	*1.23*	*1.01*–*1.51*	*0.036*	2.87	2.31–3.56	<.001	*1.31*	*1.09*–*1.56*	*0.003*
Grade												
I	Ref			Ref*			Ref			Ref*		
II	2.70	2.18–3.34	<.001	*1.42*	*1.25*–*1.63*	<*.001*	1.93	1.64–2.26	<.001	*1.24*	*1.12*–*1.37*	<*.001*
III	4.22	3.43–5.19	<.001	*1.50*	*1.30*–*1.73*	<*.001*	2.89	2.48–3.38	<.001	*1.22*	*1.09*–*1.36*	<*.001*
Lymph nodes												
Negative	Ref			Ref*			Ref			Ref*		
1–3 positive	1.94	1.71–2.21	<.001	*1.25*	*1.14*–*1.37*	<*.001*	1.59	1.43–1.77	<.001	*1.20*	*1.11*–*1.31*	<*.001*
>3 positive	5.45	4.78–6.22	<.001	*1.78*	*1.59*–*1.99*	<*.001*	3.71	3.30–4.16	<.001	*1.61*	*1.44*–*1.79*	<*.001*
Endocrine status												
ER and PR negative	Ref			Ref*			Ref					
ER/PR positive	0.56	0.50–0.63	<.001	*1.40*	*1.23*–*1.60*	<*.001*	0.56	0.50–0.62	<.001			
Histology												
Ductal	Ref			Ref*			Ref					
Lobular	1.05	0.89–1.24	0.582	*1.03*	*0.91*–*1.17*	*0.61*	1.04	0.90–1.20	0.590			
Other	0.67	0.54–0.83	<.001	*0.89*	*0.77*–*1.04*	*0.155*	0.73	0.61–0.87	<.001			
Multifocality												
No	Ref			Ref			Ref			Ref		
Yes	1.5	1.29–1.75	<.001	1.19	1.01–1.40	0.037	1.34	1.16–1.53	<.001	1.25	1.09–1.44	0.002
Surgery type												
Breast conserving	Ref			Ref			Ref					
Mastectomy	1.79	1.61–1.99	<.001	1.41	1.25–1.58	<.001	1.58	1.44–1.73	<.001			
Residual disease												
No	Ref											
Microscopic	1.16	0.87–1.53	0.311									
Chemotherapy												
No	Ref						Ref			Ref*		
Yes	1.93	1.74–2.15	<.001				1.56	1.42–1.71	<.001	*0.88*	*0.80*–*0.96*	*0.006*
Radiation therapy												
No	Ref						Ref					
Yes	1.09	0.97–1.22	0.161				0.97	0.88–1.06	0.478			
Endocrine therapy												
No	Ref						Ref					
Yes	1.47	1.32–1.63	<.001				1.12	1.03–1.23	0.012			

*LR* local recurrence, *RR* regional recurrence, *DM* distant recurrence, *HR* hazard ratio, *CI* confidence interval, *ER* oestrogen receptor, *PR* progesterone receptor, *Ref.* reference group

* time-dependent variable in analysis

**Table 3:** Prognostic factors for subsequent recurrence after previous LR or RR

Characteristic	Event after LR (*n* = 362, 102 events)						Event after RR (*n* = 148, 74 events)					
	Univariable			Multivariable			Univariable			Multivariable		
	HR	95% CI	*p*	HR	95% CI	*p*	HR	95% CI	*p*	HR	95% CI	*p*
Age (years)												
<40	Ref.			Ref			Ref			Ref		
40–49	0.9	0.44–1.85	0.772	1.06	0.50–2.24	0.889	1	0.43–2.31	0.999	0.89	0.38–2.11	0.793
50–74	0.83	0.43–1.58	0.567	1.04	0.52–2.09	0.904	1.19	0.61–2.35	0.609	1.87	0.90–3.88	0.093
≥75	1.75	0.80–3.83	0.158	1.47	0.62–3.46	0.378	0.21	0.03–1.67	0.141	0.29	0.04–2.29	0.240
Tumour size												
≤2 cm	Ref.			Ref			Ref			Ref		
2–5 cm	3.28	2.17–4.95	<.001	3.01	1.91–4.74	0.000	1.04	0.65–1.67	0.865	0.83	0.50–1.38	0.467
>5 cm	7.85	3.29–18.74	<.001	2.60	0.95–7.10	0.062	1.34	0.41–4.38	0.625	1.64	0.49–5.54	0.426
Grade												
I	Ref.			Ref			Ref					
II	2.31	1.06–5.04	0.036	1.25	0.55–2.83	0.595	0.91	0.41–2.04	0.825			
III	5.72	2.71–12.07	<.001	3.31	1.49–7.37	0.003	1.04	0.48–2.23	0.922			
Lymph nodes												
Negative	Ref.			Ref			Ref					
1–3 positive	2.98	1.92–4.64	<.001	1.94	1.20–3.14	0.007	1.07	0.62–1.82	0.816			
>3 positive	7.03	4.05–12.21	<.001	3.23	1.65–6.30	0.001	2.25	1.01–4.98	0.047			
Hormone status												
ER&PR−	Ref.			Ref*			Ref			Ref		
ER/PR+	0.41	0.27–0.62	<.001	*0.6*	*0.41*–*0.96*	*0.032*	0.58	0.36–0.92	0.021	0.6	0.31–1.13	0.111
Histology												
Ductal	Ref.			Ref			Ref					
Lobular	0.77	0.42–1.46	0.436	0.73	0.37–1.42	0.352	1.56	0.71–3.43	0.266			
Other	0.15	0.04–0.62	0.009	0.14	0.03–0.60	0.008	1.09	0.40–3.01	0.867			
Multifocality												
No	Ref.			Ref			Ref			Ref		
Yes	0.98	0.52–1.87	0.961	1.20	0.76–2.01	0.398	1.07	0.47–0.89	0.880	1.59	0.66–3.82	0.297
Surgery type												
BCS	Ref.						Ref					
Mastectomy	2.67	1.79–3.97	<.001				0.87	0.55–1.39	0.557			
Residual disease												
No	Ref.						Ref					
Microscopic	0.31	0.04–2.17	0.236				0.79	0.19–1.17	0.743			
Chemotherapy												
No	Ref.						Ref					
Yes	3.07	2.07–4.54	<.001				1.1	0.68–1.76	0.696			
Radiation therapy												
No	Ref.						Ref					
Yes	0.58	0.39–0.86	0.007				1.56	0.98–2.46	0.058			
Endocrine therapy												
No	Ref.			Ref*			Ref			Ref		
Yes	1.94	1.30–2.91	0.001	*1.60*	*1.08*–*2.42*	*0.020*	0.98	0.60–1.59	0.930	1.3	0.73–2.31	0.381
DFI (years)												
0–1.9	Ref.						Ref			Ref*		
2.0–3.9	0.58	0.37–0.91	0.02				0.98	0.57–1.68	0.930	*0.97*	*0.59*–*1.59*	*0.899*
4.0–5.9	0.35	0.19–0.65	<.001				0.7	0.36–1.36	0.298	*2.07*	*1.04*–*4.16*	*0.040*
6.0–7.9	0.15	0.05–0.41	<.001				0.58	0.25–1.33	0.198	*3.94*	*1.42*–*10.96*	*0.009*
8.0–10.0	0.29	0.10–0.82	0.02				0.25	0.03–1.83	0.170	*4.34*	*0.24*–*76.81*	*0.317*
Surgery of recurrence												
No	Ref.			Ref			Ref			Ref		
Yes	0.33	0.20–0.55	<.001	0.40	0.21–0.75	0.005	0.52	0.33–0.82	0.005	0.42	0.26–0.70	0.001
Chemotherapy of recurrence												
No	Ref.			Ref			Ref					
Yes	1.03	0.60–1.76	0.92	0.66	0.37–1.19	0.170	1.31	0.83–2.08	0.245			
Radiation therapy of recurrence												
No	Ref.			Ref			Ref					
Yes	2.02	1.37–2.98	<.001	1.51	0.96–2.39	0.076	1.4	0.87–2.26	0.169			
Endocrine therapy of recurrence												
No	Ref.						Ref			Ref		
Yes	0.69	0.46–1.04	0.07				0.61	0.38–0.98	0.040	0.64	0.36–1.17	0.148

*LR* local recurrence, *RR* regional recurrence, *HR* hazard ratio, *CI* confidence interval, *BCS* breast conserving surgery, *PR* progesterone receptor, *Ref.* reference group, *DFI* disease-free interval

* time-dependent variable in analysis

### Survival after the primary tumour and after recurrence

Ten-year survival after the primary tumour differed significantly (*p* < 0.001) with 82% for women without recurrence, compared to 61, 41 and 20% for women with local, regional or distant recurrence, respectively (Supplementary Figure S14). There was also a significant difference in survival after the first (*p* < 0.001) and second recurrence (*p* = 0.021). The ten-year survival after a first local, regional or distant recurrence was 47, 31 and 5%, respectively (Supplementary Figure S15). After a first local or regional recurrence, the ten-year survival was 21, 15 and 9% for local, regional or distant second recurrences, respectively (Supplementary Figure S16).

## Discussion

We investigated the pattern of site-specific recurrence and identified prognostic factors for first and subsequent recurrences during a follow-up of ten years using data from 9342 women treated for primary invasive breast cancer. The pattern of first recurrence was comparable for LR, RR and DM with a major peak in the second year after starting follow-up. The pattern, as well as identified prognostic factors for overall first recurrence, seemed to be dominated by the high percentage of DM. When recurrence was analysed according to site, a difference in identified prognostic factors was present. The hazard of subsequent recurrences after LR and RR both declined towards the end of follow-up. The risk of developing a second recurrence was significantly higher after RR than after LR. And after a second recurrence, more than half of the women were diagnosed with a third.

The pattern of first recurrence is consistent with previous studies [10–12, 16]. As more aggressive tumours recur earlier, individuals with these type of tumours are thereby censored, leaving only patients with tumours that grow more slowly and have more favourable characteristics. This leads to an early peak in the recurrence risk and keeps long-term recurrence rates much lower [21]. In our data, a second peak in the hazard of recurrence was present between year eight and nine. This pattern was present in the different recurrence types as well as in different subgroups (see Supplementary Figs. S1–13). However, as there were only 299 events between the years 7–10 and the 95% CIs around the hazard showed overlap, this finding is not statistically significant. Jatoi et al. [11] observed a second peak around year five and hypothesized influence from interval censoring as patients were followed clinically with regular intervals in this period. Yin et al. [12] report a second recurrence peak near the 9.5th year and attributed the bimodal pattern to tumour dormancy: a state in which tumour cells are present, but disease progression not clinically apparent [22]. Demicheli et al. [13] reason that biological characteristics are responsible for the distinctive pattern in tumour recurrence. Our findings confirm this, as patients with late recurrence (second peak) showed more favourable patient and tumour characteristics than patients with early recurrence. Besides more favourable characteristics, a previous study also found better survival for patients with longer DFIs during five years of follow-up [9]. As the follow-up period of patients with late recurrence was much shorter than follow-up for patients with early recurrence, we could not compare the amount of subsequent recurrences between the groups.

The peak in risk was most pronounced for patients with grade III tumours or negative hormone status, and more gradual in patients with ER-positive tumours. In this study, ER-positive patients received endocrine therapy for a maximum of five years. Present guidelines recommend extended endocrine treatment beyond five years [23]. This could delay or prevent most of the ER-positive recurrences that constitute the late recurrence peak. Additionally, efforts to improve adherence to endocrine therapy and the introduction of aromatase inhibitors will likely flatten the second recurrence peak [24]. In hormone receptor-positive breast cancer, late recurrences after >20 years can occur [25], as hormone-positive tumours have slower doubling times and might be suppressed for a prolonged time by endocrine therapy [26]. Also, there may be influence of immune surveillance in controlling progression [27]. Although patients with first recurrence eight to nine years after starting follow-up were more often hormone receptor positive, there was no significant difference in percentage receiving endocrine therapy when comparing patients with early and late recurrences.

Consistent with previous studies, younger age, larger primary tumour and higher grade were important prognostic factors for first recurrence [4, 8, 10]. The HRs of the prognostic factors for subsequent recurrence after LR or RR were higher than those for first recurrence, as can be explained by the higher incidence. With a highest HR of 3.7 for first recurrence (>3 positive nodes, compared N0), it is impossible to appoint one or just a few prognostic factors that can be used for risk stratification. Thrift et al. [28] state that in the case of relative risk, factors should be higher than ten for good prediction of individual risk. In the absence of a ‘perfect predictor’, multiple factors need to be taken into account for risk prediction and subsequent follow-up.

With only 33 patients at risk and 18 events after two previous LRs or RRs, it was hard to identify prognostic factors for third recurrence. The number of events determines the statistical strength of a multivariable analysis. A commonly used rule of thumb based on simulation studies [29–31], is a ratio of at least ten to twenty events per explaining variable to maintain the validity of the model. However, there are others suggesting that this rule is too conservative [32].

Strengths of this study include the large sample size, a follow-up duration of ten years and inclusion of an unselected patient cohort from the population-based NCR, representative for the majority of breast cancer patients. By extracting data in retrospect from patient medical records, time of recurrence diagnosis was determined with high accuracy. This is important in avoiding bias in the shape of hazard [33]. We used extended regression, while the proportional hazards assumption did not hold for several variables, as could be expected with long-term follow-up [34]. Changes and improvements in diagnostic procedures and treatment have resulted in a sustained decline in breast cancer recurrence [35], which means data from 2003 may not be representative for the current risk of recurrence. Because regular registration of Human Epidermal Receptor-2 (HER-2) status initiated in 2005, this prognostic factor could not be included in the analysis. However, as trastuzumab treatment for HER-2-positive patients is currently standard practice, the prognostic value of HER-2 in models is less important [4]. Inclusion of more recurrence characteristics than site and treatment, could likely result in better risk prediction of subsequent recurrence than models largely based on primary tumour characteristics. As it is hard to distinguish second primary breast cancer from contralateral recurrence, only ipsilateral and distant recurrence were taken into account in the analysis, which might have resulted in underestimation of the actual recurrence rate [36]. In addition, even though second primary tumours are of importance for follow-up, the focus of this study was on recurrence of the primary tumour and their corresponding characteristics. Although subsequent recurrence does occur in a small subset of patients diagnosed with DCIS, these were excluded from the current analysis [37, 38]. During the follow-up period, 27% of the patients died. The 10-year survival after the primary tumour, as well as after the first and second recurrence differed significantly per recurrence type. For more details on factors influencing 5-year survival after recurrence, the reader is referred to Witteveen et al. [9]. Competing risk analysis was not considered relevant as breast cancer specific mortality was assumed to be only possible after DM, and further recurrence after DM were not taken into account. Time to subsequent recurrence was counted from diagnosis of first recurrence, instead of three months afterwards, as the time until second recurrence is on average much shorter (median DFI 1.1 years, compared to 3.3 for first recurrence). This means almost half of all possible second recurrences will be diagnosed in the first year of follow-up, in which most patients already have more hospital visits due to prolonged treatment or aftercare. Also, more than 80% of the second recurrences after LR or RR, and almost 80% of third recurrences are DM, of which early detection is not aim of regular follow-up care, as earlier detection will not (yet) result in better prognosis.

This study is, to the best of our knowledge, the first analysis that takes site-specific first and also subsequent recurrence after LR and RR into account. The pattern of first recurrence was similar for LR, RR and DM with a major peak in recurrence risk around the second year post-diagnosis. The hazard of subsequent recurrence was higher if the first recurrence was RR compared to LR. As most risk factors only have modest effects, multiple risk factors need to be taken into account for risk prediction and subsequent follow-up decisions. Although the percentage of patients with first recurrence that develop a second recurrence is high, the percentage among all breast cancer patients remains very low (1.9% during ten years). As almost 50% of the second recurrences will be diagnosed in the first follow-up year, combined with the low absolute number of second recurrences, more intensive follow-up for detection of subsequent recurrence is not likely to be (cost-)effective.

## Electronic supplementary material

Below is the link to the electronic supplementary material.

Supplementary material 1 (DOCX 118 kb)
