# Supplementary material for: Patterns and predictors of first and subsequent recurrence in women with early breast cancer
Source: Breast Cancer Res Treat. 2017 Jul 4;165(3):709–20. doi: 10.1007/s10549-017-4340-3 (PMC5602040; doi:10.1007/s10549-017-4340-3)
Supplement: Supplementary file 1 — Supplementary material 1 (DOCX 118 kb) [file 10549_2017_4340_MOESM1_ESM.docx]

**Supplementary material**

**Fig. S1** Hazard of recurrence over time by age category

| Hazard | 1 | 2 | 3 | 4 | 5 | 6 | 7 | 8 | 9 | 10 |
| --- | --- | --- | --- | --- | --- | --- | --- | --- | --- | --- |
| <40 | .0537 | .0741 | .0639 | .0253 | .0328 | .0317 | .0352 | .0293 | .0227 | .0077 |
| 40-49 | .0307 | .0405 | .0338 | .0308 | .0229 | .0236 | .0181 | .0151 | .0126 | .0186 |
| 50-75 | .0193 | .0340 | .0293 | .0252 | .0247 | .0167 | .0200 | .0154 | .0119 | .0176 |
| >75 | .0350 | .0455 | .0458 | .0236 | .0343 | .0256 | .0170 | .0077 | .0246 | .0158 |

**Fig. S2** Hazard of recurrence over time by primary tumour size

| Hazard | 1 | 2 | 3 | 4 | 5 | 6 | 7 | 8 | 9 | 10 |
| --- | --- | --- | --- | --- | --- | --- | --- | --- | --- | --- |
| ≤2cm | .0112 | .0231 | .0256 | .0191 | .0193 | .0136 | .0160 | .0132 | .0115 | .0134 |
| >2-5cm | .0466 | .0626 | .0476 | .0384 | .0367 | .0309 | .0282 | .0203 | .0173 | .0208 |
| >5cm | .0621 | .0991 | .0621 | .0463 | .0443 | .0296 | .0313 | .0265 | .0000 | .0655 |

**Fig. S3** Hazard of recurrence over time by primary tumour nodal involvement

| Hazard | 1 | 2 | 3 | 4 | 5 | 6 | 7 | 8 | 9 | 10 |
| --- | --- | --- | --- | --- | --- | --- | --- | --- | --- | --- |
| 0 nodes | .0132 | .0236 | .0251 | .0220 | .0199 | .0138 | .0172 | .0123 | .0094 | .0145 |
| 1-3 nodes | .0303 | .0407 | .0349 | .0228 | .0306 | .0256 | .0240 | .0212 | .0210 | .0195 |
| >3 nodes | .0830 | . 1314 | .0907 | .0675 | .0546 | .0504 | .0340 | .0262 | .0254 | .0309 |

**Fig. S4** Hazard of recurrence over time by primary tumour grade

| Hazard | 1 | 2 | 3 | 4 | 5 | 6 | 7 | 8 | 9 | 10 |
| --- | --- | --- | --- | --- | --- | --- | --- | --- | --- | --- |
| I | .0060 | .0101 | .0155 | .0118 | .0128 | .0100 | .0148 | .0087 | .0096 | .0172 |
| II | .0172 | .0303 | .0293 | .0287 | .0317 | .0207 | .0223 | .0175 | .0172 | .0188 |
| III | .0507 | .0738 | .0578 | .0332 | .0312 | .0258 | .0182 | .0168 | .0108 | .0134 |

**Fig. S5** Hazard of recurrence over time by primary tumour hormone status

| Hazard | 1 | 2 | 3 | 4 | 5 | 6 | 7 | 8 | 9 | 10 |
| --- | --- | --- | --- | --- | --- | --- | --- | --- | --- | --- |
| ER&PR- | .0814 | .0989 | .0597 | .0339 | .0231 | .0185 | .0105 | .0079 | .0061 | .0072 |
| ER/PR+ | .0135 | .0267 | .0290 | .0243 | .0270 | .0203 | .0223 | .0178 | .0155 | .0194 |

**Fig. S6** Hazard of recurrence over time by primary tumour multifocality

| Hazard | 1 | 2 | 3 | 4 | 5 | 6 | 7 | 8 | 9 | 10 |
| --- | --- | --- | --- | --- | --- | --- | --- | --- | --- | --- |
| No | .0252 | .0365 | .0326 | .0255 | .0269 | .0189 | .0190 | .0155 | .0135 | .0164 |
| Yes | .0319 | .0627 | .0432 | .0351 | .0274 | .0274 | .0271 | .0187 | .0161 | .0150 |

**Fig. S7** Hazard of recurrence over time by primary tumour residue

| Hazard | 1 | 2 | 3 | 4 | 5 | 6 | 7 | 8 | 9 | 10 |
| --- | --- | --- | --- | --- | --- | --- | --- | --- | --- | --- |
| No | .0246 | .0387 | .0334 | .0248 | .0257 | .0199 | .0201 | .0155 | .0132 | .0163 |
| Microscopic | .0192 | .0433 | .0316 | .0370 | .0314 | .0165 | .0259 | .0136 | .0143 | .0202 |

**Fig. S8** Hazard of recurrence over time by primary tumour histology

| Hazard | 1 | 2 | 3 | 4 | 5 | 6 | 7 | 8 | 9 | 10 |
| --- | --- | --- | --- | --- | --- | --- | --- | --- | --- | --- |
| Ductal | .0285 | .0423 | .0355 | .0263 | .0254 | .0196 | .0189 | .0148 | .0134 | .0168 |
| Lobular | .0150 | .0300 | .0304 | .0307 | .0334 | .0277 | .0292 | .0223 | .0192 | .0234 |
| Other | .0151 | .0240 | .0274 | .0194 | .0200 | .0138 | .0216 | .0150 | .0094 | .0129 |

**Fig. S9** Hazard of recurrence over time by primary tumour surgery

| Hazard | 1 | 2 | 3 | 4 | 5 | 6 | 7 | 8 | 9 | 10 |
| --- | --- | --- | --- | --- | --- | --- | --- | --- | --- | --- |
| Breast conserving | .0162 | .0256 | .0267 | .0228 | .0229 | .0172 | .0180 | .0144 | .0134 | .0157 |
| Mastectomy | .0374 | .0565 | .0440 | .0307 | .0297 | .0237 | .0236 | .0174 | .0140 | .0192 |

**Fig. S10** Hazard of recurrence over time by primary tumour chemotherapy

| Hazard | 1 | 2 | 3 | 4 | 5 | 6 | 7 | 8 | 9 | 10 |
| --- | --- | --- | --- | --- | --- | --- | --- | --- | --- | --- |
| No | .0190 | .0278 | .0268 | .0235 | .0251 | .0177 | .0180 | .0140 | .0126 | .0165 |
| Yes | .0383 | .0605 | .0483 | .0311 | .0270 | .0240 | .0244 | .0184 | .0155 | .0181 |

**Fig. S11** Hazard of recurrence over time by primary tumour radiation therapy

| Hazard | 1 | 2 | 3 | 4 | 5 | 6 | 7 | 8 | 9 | 10 |
| --- | --- | --- | --- | --- | --- | --- | --- | --- | --- | --- |
| No | .0306 | .0403 | .0380 | .0231 | .0232 | 0207. | .0209 | .0154 | .0128 | .0160 |
| Yes | .0232 | .0386 | .0322 | .0276 | .0270 | .0195 | .0200 | .0157 | .0140 | .0176 |

**Fig. S12** Hazard of recurrence over time by primary tumour endocrine therapy

| Hazard | 1 | 2 | 3 | 4 | 5 | 6 | 7 | 8 | 9 | 10 |
| --- | --- | --- | --- | --- | --- | --- | --- | --- | --- | --- |
| No | .0323 | .0406 | .0336 | .0255 | .0255 | .0165 | .0161 | .0113 | .0093 | .0141 |
| Yes | .0169 | .0374 | .0348 | .0271 | .0302 | .0245 | .0260 | .0215 | .0196 | .0212 |

**Fig. S13** Hazard of recurrence over time by primary tumour axillary lymph node dissection

| Hazard | 1 | 2 | 3 | 4 | 5 | 6 | 7 | 8 | 9 | 10 |
| --- | --- | --- | --- | --- | --- | --- | --- | --- | --- | --- |
| No | .0372 | .0531 | .0412 | .0301 | .0306 | .0267 | .0226 | .0184 | .0187 | .0195 |
| Yes | .0123 | .0234 | .0263 | .0218 | .0205 | .0127 | .0178 | .0127 | .0084 | .0146 |

**Fig. S14** Survival after the primary tumour for women without recurrence and women with LR, RR or DM.

^Abbreviations: LR = local recurrence, RR = regional recurrence, DM = distant metastasis^

**Fig. S15** Survival after the first recurrence for women with LR, RR or DM.

^Abbreviations: LR = local recurrence, RR = regional recurrence, DM = distant metastasis^

**Fig. S16** Survival after the second recurrence for women with LR, RR or DM, after a previous LR or RR.

^Abbreviations: LR = local recurrence, RR = regional recurrence, DM = distant metastasis^
